# Shoulder Subluxation Pain as a Secondary Indication for Trapezius to Deltoid Transfer

**DOI:** 10.1055/s-0038-1676786

**Published:** 2018-12-31

**Authors:** Andrew I. Elkwood, Michael I. Rose, Matthew R. Kaufman, Tushar R. Patel, Russell L. Ashinoff, Adam Saad, Lisa F. Schneider, Eric G. Wimmers, Hamid Abdollahi, Deborah Yu

**Affiliations:** 1 The Institute for Advanced Reconstruction, Shrewsbury, New Jersey, United States

**Keywords:** trapezius, deltoid, muscle transfer, shoulder subluxation, pain

## Abstract

Brachial plexus injuries can be debilitating. We have observed that manual reduction of the patients' shoulder subluxation improves their pain and have used this as a second reason to perform the trapezius to deltoid muscle transfer beyond motion. The authors report a series of nine patients who all had significant improvement of pain in the shoulder girdle and a decrease in pain medication use after a trapezius to deltoid muscle transfer. All patients were satisfied with the outcomes and stated that they would undergo the procedure again if offered the option. The rate of major complications was low. The aim is not to describe a new technique, but to elevate a secondary indication to a primary for the trapezius to deltoid transfer beyond improving shoulder function: pain relief from chronic shoulder subluxation.

## Introduction

Brachial plexus injuries can be devastating. For optimal recovery, the current recommendations suggest treatment within 6 months from the time of injury. 1 When a patient presents with brachial plexus injuries, there are three main goals of treatment: restoration of function, restoration of sensibility, and pain relief. Neurotization is almost always the first choice if possible. Tendon transfer is the initial reconstructive step in patients who fail to improve shoulder function after brachial plexus nerve reconstruction or in patients who present in a delayed fashion outside the window for nerve repair or transfer. Tendon transfer is performed to restore active shoulder external rotation and possibly even flexion, abduction, and internal rotation. 2 There are many descriptions of muscle transfers to restore function and stability of the shoulder. 3 4 5 6 7 Muscles that have been used for transfer include the trapezius, pectoralis major, teres major, latissimus dorsi, and combined biceps and triceps. 3 The solution is based upon the individual patient's nerve symptoms and potential donors or associated nerve pain. Another option that has been offered in patients with brachial plexus injury is shoulder arthrodesis. 8 9 This procedure can result in improvement in active function postoperatively as the scapula still contributes to motion. 8 However, complications after the procedure are frequent, reported to be as high as 28%. 8 10

Patients with brachial plexus injuries present with two types of pain: (1) phantom pain from nerve root avulsion or associated nerve pain and (2) chronic subluxation pain in the shoulder. Most of the muscles around the shoulder are involved in the injury, leaving few muscles available for tendon transfer. The trapezius muscle is typically spared after pan-plexopathy because of its innervation by the spinal accessory nerve and contributions of C3 and C4. 2 As part of the reconstruction of the brachial plexus injury, there have been many descriptions of the trapezius to deltoid transfer method to restore the function of the nonfunctioning deltoid. 11 12 In the pan-brachial plexus injury, there are no other muscles in the plexus available beyond the trapezius to use. With this trapezius-to-deltoid transfer, there is help with axillary hygiene as well. More valuable, however, is the resulting pain relief.

We have observed that manual reduction of patients' shoulder subluxation often improves their pain. This observation provides a second reason to perform the muscle transfer beyond motion. Despite undergoing brachial plexus reconstruction, some patients request an elective amputation. With such amputation, pain was only improved in five of nine patients (55.6%), as reported by Maldonado et al. 13 Rather than resort to amputation for pain relief, we present the trapezius-to-deltoid transfer procedure for the primary indication for pain relief from chronic subluxation of the shoulder pain, bringing forth an additional indication beyond the original one to improve shoulder abduction.

## Patients and Methods

### Patients

Patients were considered candidates for this procedure if there was pain relief on manual reduction of the shoulder subluxation in the office, which would provide a second reason for the surgery beyond motion. Shoulder shrug test was performed to ensure a functioning trapezius muscle, a prerequisite for the tendon transfer surgery. All patients had a brachial plexopathy involving the deltoid muscle. The procedures were timed to take place after the window for neurotization. A total of nine patients underwent a trapezius to deltoid transfer ( Table 1 ).

**Table 1:** History of injury by patient

Unique ID	Cause of injury	Duration from injury to surgery (mo)	Previous surgeries	Duration of follow-up (mo)
1	Fall	12	Surgery for distal radial fracture following injury	10
2	Industrial accident	7	Arteriovenous subclavian reconstruction and nerve reconstruction (details unavailable)	27.5
3	Cervical spine surgery	39	Neck surgery following cervical spine surgery	5
4	Motorcycle accident	22	Brachial plexus repair using nerve grafts to musculocutaneous nerve, median nerve, axillary nerve, and radial nerve (intact trapezius function)	57
5	Motorcycle accident	10	Nerve graft to median and axillary nerves, carpal tunnel release, repair of pectoralis minor and major (intact shoulder shrug)	43
6	Possible radiation plexopathy	24	N/A	60
7	Motor vehicle accident	600	N/A	6
8	Motorcycle accident	29	Brachial plexus repair using nerve grafts (intact shoulder shrug)	118
9	Work-related accident	36	Brachial plexus exploration and nerve graft (intact shoulder shrug)	14.5

### Operative Technique

**Fig. 1:**
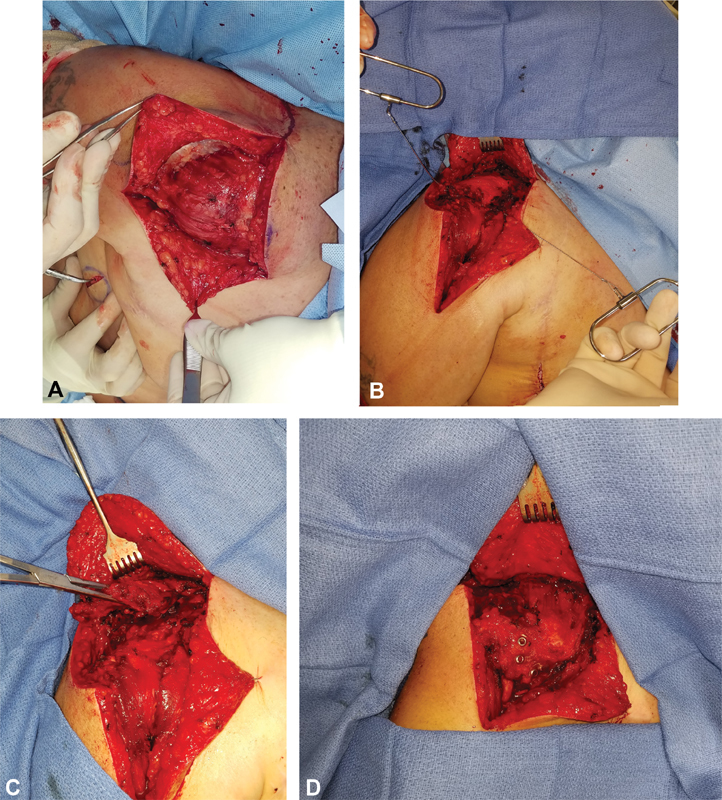
Intraoperative photographs depicting surgical technique. A U-shaped incision with a vertical extension is made surrounding the acromial joint and over the humerus inferiorly ( **A** ). Using a Gigli saw, a transverse ostectomy of the acromioclavicular joint is performed, maintaining the soft tissue over the anterior acromion process ( **B** ). The trapezius is elevated off the scapula and the clavicle with care to avoid injury of the spinal accessory nerve ( **C** ). Three cannulated screws are placed through the acromion process to mobilize it anterolaterally and as far distally as possible on the humerus while the arm is in an abducted and slightly rotated position ( **D** ).

We are describing our operative technique, which is not meant to be unique from those previously described in the literature. A U-shaped incision with a vertical extension was made surrounding the acromial joint and over the humerus inferiorly ( Fig. 1A ). The deltoid muscle was incised transversely and vertically to elevate it from the acromial joint. The deltoid was dissected anteriorly and posteriorly down to the level of the humerus. The anterolateral aspect of the humerus was stripped of its periosteum. Using a Midas Rex saw (Medtronic, Minneapolis, Minnesota, United States), a partial humeral ostectomy was performed to partially remove the outer table of the cortex to provide better bone-to-bone apposition. Dissection was then carried under the acromion process. Using a Gigli saw, a transverse ostectomy of the acromioclavicular joint was performed, maintaining the soft tissue over the anterior acromion process ( Fig. 1B ). The trapezius was elevated off the scapula and the clavicle with care to avoid injury of the spinal accessory nerve. The trapezius was dissected approximately 10 to 15 cm proximally ( Fig. 1C ).

With the aid of fluoroscopy, three 4.5-mm cannulated screws were placed through the acromion process to mobilize it anterolaterally and as far distally as possible on the humerus while the arm was in an abducted and slightly rotated position ( Fig. 1D ). In some of the procedures, a rongeur was used to harvest bone graft from the remnants of the acromion process to facilitate fusion.

The deltoid was then transferred into the trapezius muscle using modified figure-of-eight #0 PDS suture (Ethicon, Somerville, New Jersey, United States). Local anesthetic for postoperative anesthesia was injected into the surrounding muscle and incision. The patient was placed in a gunslinger splint in maximal shoulder abduction for 6 weeks.

## Results

**Fig. 2:**
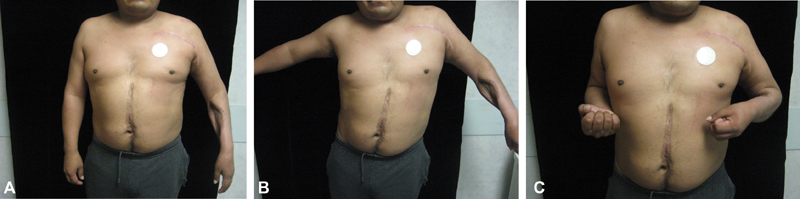
Photograph of patient demonstrating postoperative shoulder range of motion.

Six males and three females underwent the procedure described above. The patients' age ranged from 22 to 63 years (median: 39 years). Body mass index (BMI) of the patients ranged from 19.7 to 33.2 (median BMI: 27.1). The follow-up ranged from 5 to 118 months (median: 27.5 months). All patients (100%) had significant improvement in pain in the shoulder girdle. Fifty percent of patients who were taking pain medication prior surgery no longer required medication. Out of the remaining 50% of patients, two-thirds required less medication. All patients were happy with the outcomes and stated that they would undergo the procedure again if offered the option. Only seven of nine patients had data regarding postoperative shoulder abduction, two were lost to follow-up. Of those seven patients, the range of shoulder abduction was from 45 to 90 degrees (median: 45 degrees) ( Fig. 2 ). There were two patients that had complications related to the transfer, necessitating additional procedure(s), including drainage of seroma, debridement of necrotic tissue, and removal of eroding suture. Following intervention, no additional complications were reported. Two other patients had minor complications that were treated with local wound care (skin breakdown and superficial wound infection). Patients were also satisfied with the aesthetic outcome, both the incision site appearance and the appearance with improved arm position. The results also allowed patients to maintain improved hygiene and those with candidiasis preoperatively had their infection resolved postoperatively.

## Discussion

In brachial plexus injuries, neurotization or primary nerve reconstruction is the gold standard in restoration of function. Sulaiman et al demonstrated that nerve transfers for reconstruction of brachial plexus injuries could result in excellent recovery of elbow and shoulder function. The accessory nerve is often utilized for the treatment of brachial plexus injuries. Sulaiman et al also reported that shoulder abduction recovery to Medical Research Council grade 3 after spinal accessory to suprascapular and/or thoracodorsal to axillary nerve transfer was 95 and 36%, respectively. 14 Chen et al reported transferring a triceps branch of the radial nerve to the axillary nerve in patients without deltoid function (but preserved triceps strength) and found that both the neurolysis alone and the nerve transfer groups had significant improvements in deltoid strength and shoulder abduction from a mean of 63 to 127 degrees. 15 When patients present outside the window for neurotization or fail to have shoulder function improvement after brachial plexus nerve reconstruction, tendon transfer is the initial reconstructive step. Few muscles are available after the injury for tendon transfer. 2 There have been several descriptions of the trapezius-to-deltoid transfer method for the restoration of deltoid function. 12 This transfer has been performed for many years with the goal of return of function. We are presenting an additional indication for performing the trapezius-to-deltoid transfer beyond a gain in function of shoulder abduction: pain relief from chronic shoulder subluxation.

We observed that manual reduction of our brachial plexus patients' shoulder subluxation improved their pain. All patients reported significant improvement of pain in the shoulder. The need for pain medication decreased in 33% of patients, while 50% discontinued use of all pain medication. Seven of the nine patients had documented shoulder abduction of at least 45 degrees (range: 45–90 degrees). The shoulder joint is unique in that the surrounding musculature must be functional to maintain reduction in the joint. Paralysis of the surrounding muscles will lead to subluxation, which is different from other joints in the body, i.e., the knee.

Limitations to our report include the retrospective design of the study and small sample size heterogeneity in patient population. Most patients also had other procedures performed in the past with limited success and persistent shoulder subluxation. It is unlikely that these additional procedures would have addressed the chronic shoulder subluxation pain.

Our results demonstrate that the trapezius-to-deltoid transfer may be considered in patients with shoulder subluxation pain with relief upon reduction as a primary reason for performing the surgery. The benefits of the procedure outweighed the potential complications with significant reduction in pain.

## Conclusion

Our article elevates an additional primary indication for the trapezius-to-deltoid transfer beyond improving shoulder function: pain relief from chronic shoulder subluxation.
